# Changes in REDD1, REDD2, and atrogene mRNA expression are prevented in skeletal muscle fixed in a stretched position during hindlimb immobilization

**DOI:** 10.1002/phy2.246

**Published:** 2014-02-25

**Authors:** Andrew R. Kelleher, Bradley S. Gordon, Scot R. Kimball, Leonard S. Jefferson

**Affiliations:** 1 Department of Cellular and Molecular Physiology, The Pennsylvania State University College of Medicine, Hershey, Pennsylvania

**Keywords:** Casting, Ddit4, Ddit4l, disuse atrophy, mTORC1

## Abstract

Immobilized skeletal muscle fixed in a shortened position displays disuse atrophy, whereas when fixed in a stretched position it does not (Goldspink, D. F. (1977) *J Physiol* 264, 267–282). Although significant advances have been made in our understanding of mechanisms involved in development of atrophy in muscle fixed in a shortened position, little is known about why mass is maintained when muscle is immobilized in a stretched position. In the present study, we hypothesized that skeletal muscle immobilized in a stretched position would be protected from gene expression changes known to be associated with disuse atrophy. To test the hypothesis, male Sprague‐Dawley rats were anesthetized using isoflurane and subjected to unilateral hindlimb immobilization for 3 days with the soleus fixed in either a shortened or stretched position. All comparisons were made to the contralateral nonimmobilized muscle. Soleus immobilized in a shortened position exhibited disuse atrophy, attenuated rates of protein synthesis, attenuated mTORC1 signaling, and induced expression of genes for REDD1, REDD2, MAFbx, and MuRF1. In contrast, immobilization of the soleus in a stretched position prevented these changes as it exhibited no difference in muscle mass, rates of protein synthesis, mTORC1 signaling, or expression of genes encoding REDD1, MAFbx or MuRF1, with REDD2 expression being reduced compared to control. In conclusion, fixed muscle length plays a major role in immobilization‐induced skeletal muscle atrophy whereby placing muscle in a shortened position leads to induction of gene expression for REDD1, REDD2, and atrogenes.

## Abstract (precis)

e00246

Immobilized skeletal muscle fixed in a shortened position displays disuse atrophy, whereas when it is fixed in a stretched position it does not. Using a rat model of unilateral hindlimb immobilization, we tested the hypothesis that skeletal muscle immobilized in a stretched position is protected from gene expression changes known to be associated with disuse atrophy. The results demonstrate that fixed muscle length plays a major role in immobilization‐induced skeletal muscle atrophy whereby placing muscle in a stretched position prevents the induction of gene expression for REDD1, REDD2, and atrogenes that is observed in muscle placed in a shortened position.

## Introduction

Prior reports from two separate laboratories showed differences in the amount of disuse atrophy (the loss of skeletal muscle mass due to unloading and physical inactivity [Stein and Wade 2005; Bodine 2013]) when hindlimb muscles were immobilized in either a shortened or stretched position (Booth 1977; Goldspink 1977). In these reports, disuse atrophy of the soleus was observed when a rat ankle joint was immobilized for 7 days in full plantarflexion (soleus muscle placed in a shortened position). In contrast, no disuse atrophy of the soleus was observed when a rat ankle joint was immobilized for 7 days in full dorsiflexion (soleus placed in a stretched position). In association with the observed patterns for muscle mass, protein synthesis was attenuated in the soleus immobilized in plantarflexion, but not in soleus immobilized in dorsiflexion (Goldspink 1977). Muscle atrophy and attenuated rates of protein synthesis were also reduced or absent in other disuse models in which skeletal muscle was immobilized in a stretched position, including hindlimb unloading and denervation (Goldspink 1978b; Loughna et al. 1986). However, the mechanism(s) through which immobilization in a stretched position acts to prevent disuse atrophy is currently unknown.

The loss of muscle mass in response to disuse occurs due to an imbalance in protein turnover (Phillips et al. 2009). Recent scientific advances have identified the cellular signaling pathways that modulate rates of protein synthesis and protein degradation in skeletal muscle. The rate of skeletal muscle protein synthesis is modulated in part by the mechanistic target of rapamycin complex 1 (mTORC1) signaling pathway (for review see [Gordon et al. 2013]). Hormones, nutrients, and exercise activate mTORC1 signaling leading to phosphorylation and activation of 70 kDa ribosomal protein S6 kinase 1 (p70S6K1). Consequently, activation of p70S6K1 promotes mRNA translation initiation, protein synthesis, and muscle hypertrophy (Kimball and Jefferson 2010). Phosphorylation of p70S6K1 has been reported to be attenuated in skeletal muscle in response to hindlimb immobilization, unloading, and bed rest (You et al. 2010; Bajotto et al. 2011; Drummond et al. 2012). Recently, we characterized an animal model of hindlimb immobilization in which rates of skeletal muscle protein synthesis and mTORC1 signaling were repressed within 24 h of immobilization (Kelleher et al. 2013). The attenuation in mTORC1 signaling occurred in parallel with an induction of gene expression for two repressors of the pathway, regulated in development and DNA‐damage response (REDD) 1 and REDD2.

In addition to repressed protein synthesis, enhanced protein degradation also contributes to the development of disuse atrophy. In part, the rate of protein degradation is modulated through the ubiquitin‐proteasome pathway (for review see [Sandri 2013]). During atrophy, it is believed that proteasome‐mediated degradation of skeletal muscle protein is regulated in part by expression of “atrogenes”; two muscle‐specific E3 ubiquitin ligases known as MAFbx/atrogin‐1 (Muscle Atrophy F‐box) and MuRF1 (Muscle Ring Finger 1) (Bodine 2013). Induction of gene expression for both MuRF1 and MAFbx has been observed in skeletal muscle in response to hindlimb immobilization and hindlimb unloading (Bodine et al. 2001; Senf et al. 2008; Baptista et al. 2010).

The purpose of the present study was to gain an understanding of the role of fixed muscle length in the regulation of protein synthesis and degradation in the soleus of an immobilized rat hindlimb. We hypothesized that the soleus placed in a stretched position would not exhibit disuse atrophy and would be protected from changes in gene expression known to be associated with disuse atrophy.

## Methods

### Animals

Adult (8–9 week; 250–350 g) male Sprague‐Dawley rats (Charles River Laboratories, Wilmington, MD) were housed in wire cages in a temperature‐ (25°C) and light‐controlled environment. Rats were provided rodent chow (Harlan‐Teklad 8604, Indianapolis, IN) and water *ad libitum*. Animal facilities and experimental protocols were submitted to, and approved by, the Institutional Animal Care and Use Committee of The Pennsylvania State University College of Medicine.

### Experimental design

Twenty rats were anesthetized with isoflurane inhalation (2.5%) and subjected to unilateral hindlimb immobilization. Ten animals had the ankle joint of one hindlimb immobilized in full plantarflexion, which placed the soleus in a shortened position, as described previously (Kelleher et al. 2013). Another 10 animals had the ankle joint of one hindlimb immobilized in full dorsiflexion, which placed the soleus in a stretched position (Goldspink 1977). The hindlimbs were immobilized for 3 days prior to removal of the soleus for subsequent analysis. An equal number of rats from the two experimental groups were processed on the days of tissue harvest. All rats were fasted overnight (18 h), but allowed free access to water. On tissue harvest days, rats were anesthetized using isoflurane and remained anesthetized for the remainder of the procedure. Immobilization for 3 days was selected for all analysis based on our previous studies showing that disuse atrophy was manifest at this time point in this experimental model (Kelleher et al. 2013).

### Sample preparation and immunoblot procedure

All rats were injected intravenously with puromycin and the soleus muscles were collected, processed, and subjected to protein immunoblot analysis as described previously (Kelleher et al. 2013). PVDF membranes were incubated with primary antibodies recognizing p70S6K1 phosphorylated on the specific residue, Thr^389^, from Cell Signaling Technology Inc (Danvers, MA). Additional blots were probed with antibodies against p70S6K1 from Bethyl Laboratories, Inc (Montgomery, TX). Protein synthesis was measured by incorporation of puromycin into peptide chains (Goodman et al. 2011) using a mouse monoclonal antipuromycin antibody generated in‐house (1 *μ*g/mL in Tris‐buffered saline) (Kelleher et al. 2013). An equal amount of sample (20 *μ*g protein) was loaded in each well and equal loading was verified by staining with Red Ponceau. Blots were developed using a FluorChem M Multifluor System (ProteinSimple, San Jose, CA) and analyzed using AlphaView (ProteinSimple) and Genetools (Syngene, Cambridge, MA) software.

### Measurement of mRNA expression

RNA was isolated using the TRIzol method (Life Technologies, Grand Island, NY) from soleus homogenates and prepared for real time polymerase chain reaction as previously described (Kelleher et al. 2013). Primers were purchased from Applied Biosystems including: Ddit4 (REDD1) (Assay ID: Rn01433735_g1); Ddit4l (REDD2) (Rn00589659_g1); and Tbp (TATA Binding Protein) (Rn01455646_m1). Tbp mRNA expression was used for an internal control as its expression did not change across a 3‐day period of immobilization.

### Statistical analysis

Results from individual studies (*n *=**5 per group) were replicated in two independent experiments. Results from the immobilized hindlimb soleus are presented as a percentage of the nonimmobilized hindlimb soleus ± SEM. For each of the variables measured, no differences were observed in soleus muscles from the nonimmobilized hindlimbs regardless of experimental group. Outliers were determined using Grubb's test (alpha level 0.05) and excluded from further analysis. Paired *t* tests were used to compare differences between soleus muscles from immobilized and nonimmobilized hindlimbs. All comparisons were performed using GraphPad Software. Differences between groups were considered significant at *P *<**0.05.

## Results

### Effect of immobilization on muscle mass and protein synthesis

**Figure 1.**
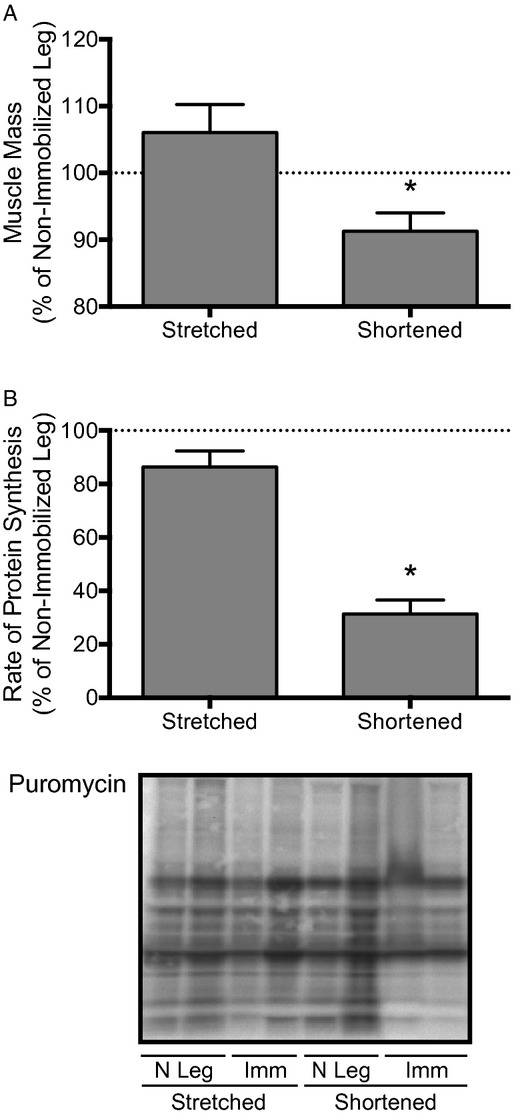
The mass and rates of protein synthesis in the soleus are reduced only when the muscle is immobilized in a shortened position. Rates of protein synthesis were measured by the amount of puromycin incorporated into protein as assessed by immunoblot analysis. Rats had one hindlimb immobilized for 3 days in a position to place the soleus muscle in either a stretched or shortened position (Imm: Immobilized; N leg = Nonimmobilized limb). Bars represent (A) the mean mass of the soleus, and (B) the mean rates of protein synthesis in the soleus from an immobilized hindlimb expressed as a percentage of the values from the soleus from the contralateral nonimmobilized hindlimb. Data are mean percentages ± SEM,* n *=**10 rats/group. *P <**0.05 versus soleus from the nonimmobilized limb.

As illustrated in Fig. 1A, immobilization of soleus in a stretched position prevented the atrophy observed in the soleus immobilized in a shortened position, (i.e. muscle mass was significantly reduced (*P *<**0.05) in soleus immobilized in a shortened position when expressed relative to soleus from the nonimmobilized hindlimb). No difference in protein content (mg/g) was observed between muscles from immobilized and nonimmobilized hindlimbs (data not shown).

To investigate the cause of the observed differences in muscle mass, rates of protein synthesis were measured in the soleus after 3 days of immobilization. As illustrated in Fig. 1B, immobilization of soleus in a stretched position prevented the reduction in rates of protein synthesis observed in the soleus immobilized in a shortened position (i.e. rates of protein synthesis were reduced approximately 70% in soleus immobilized in a shortened position when expressed relative to that of the soleus from the nonimmobilized hindlimb).

### Effect of immobilization on regulation of mTORC1 signaling

**Figure 2.**
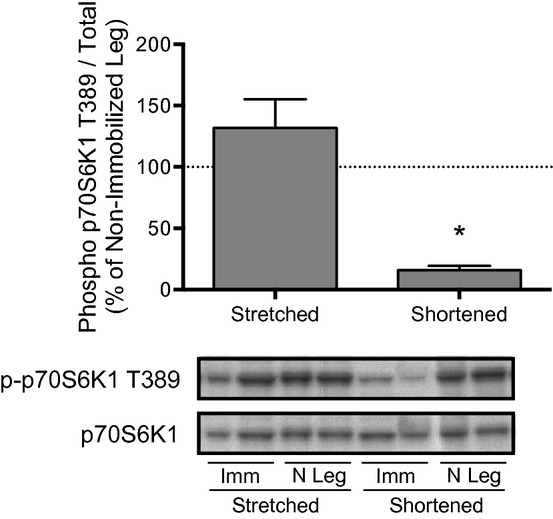
Phosphorylation of p70S6K1 at Thr389 in the soleus is reduced only when the muscle is immobilized in a shortened position. Phosphorylation of p70S6K1 at Thr389 was assessed by protein immunoblot analysis. Rats had one hindlimb immobilized for 3 days in a position to place the soleus in either a stretched or shortened position (Imm: Immobilized; N leg = Nonimmobilized limb). Bars represent the mean p70S6K1 phospho/total protein ratio in the soleus from an immobilized hindlimb expressed as a percentage of that in the soleus from the contralateral nonimmobilized hindlimb. No changes in total p70S6K1 expression were observed in any group across 3 days of hindlimb immobilization. Data are mean percentages ± SEM,* n *=**10 rats/group. Imm *P <**0.05 versus soleus from the nonimmobilized limb.

To gain an understanding of potential molecular events contributing to the changes in protein synthesis during hindlimb immobilization, mTORC1 signaling was assessed after 3 days of immobilization. An analysis of the phosphorylation state of p70S6K1 at Thr389 (Fig. 2) demonstrated that immobilization of soleus in a stretched position prevented the reduction in mTORC1 signaling observed in soleus immobilized in a shortened position (i.e. phosphorylation of p70S6K1 at Thr389 was reduced approximately 85% in soleus immobilized in a shortened position when expressed relative to that of the soleus from the nonimmobilized hindlimb).

**Figure 3.**
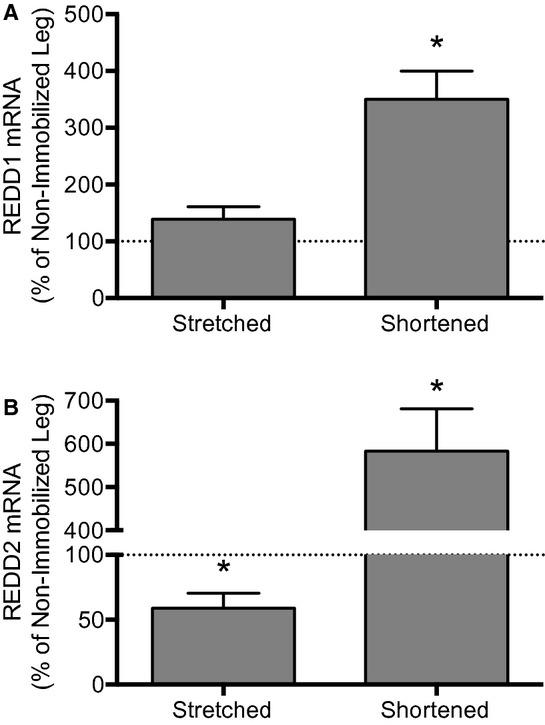
REDD1 and REDD2 mRNA expression in the soleus is induced only when the muscle is immobilized in a shortened position. REDD1 and REDD2 mRNA expression were assessed by Taqman gene expression assay. Rats had one hindlimb immobilized for 3 days in a position to place the soleus in either a stretched or shortened position. Bars represent the mean (A) REDD1 mRNA‐, or (B) REDD2 mRNA‐to‐Tbp mRNA ratio in the soleus from an immobilized hindlimb expressed as a percentage of the same ratio in the soleus from the contralateral nonimmobilized hindlimb. No changes in Tbp gene expression were observed in any group across 3 days of hindlimb immobilization. Data are mean percentages ± SEM, *n *=**10 rats/group. *P <**0.05 versus soleus from the non‐immobilized limb.

Attenuation of mTORC1 signaling following hindlimb immobilization in plantarflexion is associated with induced expression of the genes encoding the mTORC1 repressors REDD1 and REDD2 (Kelleher et al. 2013). In the present study, immobilization of the soleus in a stretched position prevented the induction of both REDD1 (Fig. 3A) and REDD2 (Fig. 3B) mRNA expression that were observed in soleus immobilized in a shortened position (i.e. mRNA expression of REDD1 and REDD2, both of which repress mTORC1 signaling through activation of the tuberous sclerosis complex consisting of TSC1 and TSC2 (Sofer et al. 2005; Miyazaki and Esser 2009), was enhanced approximately 250% and nearly 500%, respectively, in soleus immobilized in a shortened position when expressed relative to that of the soleus from the nonimmobilized hindlimb). Interestingly, soleus immobilized in a stretched position displayed a 40% reduction in REDD2 expression (Fig. 3B). Overall, these results are consistent with a model in which immobilization of soleus in a stretched position prevents the induction of REDD1 and REDD2 expression that are responsible for attenuated mTORC1 signaling and protein synthesis in skeletal muscle immobilized in a shortened position.

### Effect of immobilization on atrogene expression

**Figure 4.**
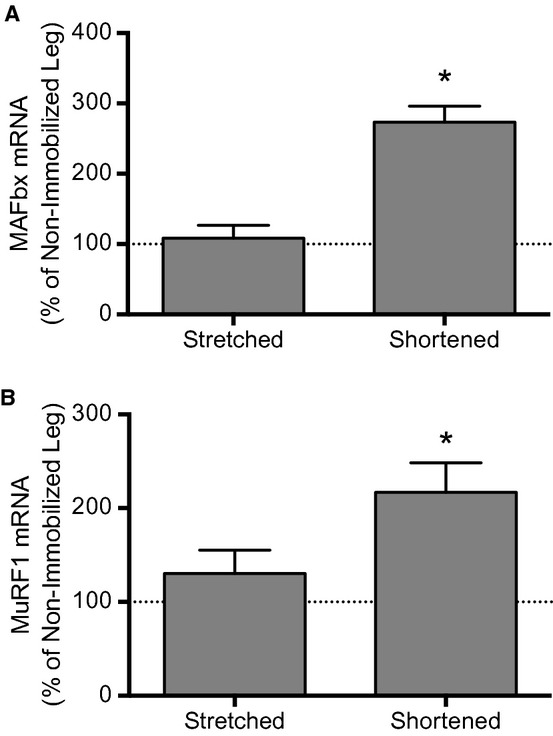
Atrogene expression is induced in the soleus only when the muscle is immobilized in a shortened position. Atrogene mRNA expression was assessed by Taqman gene expression assay. Rats had one hindlimb immobilized for 3 days in a position to place the soleus muscle in either a stretched or shortened position. Bars represent the mean (A) MAFbx mRNA‐, or (B) MuRF1 mRNA‐to‐Tbp mRNA ratio in the soleus from an immobilized hindlimb expressed as a percentage of the same ratio in the soleus from the contralateral nonimmobilized hindlimb. No changes in Tbp gene expression were observed in any group across 3 days of hindlimb immobilization. Data are mean percentages ± SEM,* n *=**10 rats/group. *P <**0.05 versus soleus from the nonimmobilized limb.

Next, we assessed mRNA expression of the E3 ubiquitin ligases, MAFbx and MuRF1, as markers of proteasome‐mediated degradation of skeletal muscle proteins during atrophy (Bodine 2013). An analysis of mRNA expression of MAFbx (Fig. 4A) and MuRF1 (Fig. 4B) demonstrated that immobilization of soleus in a stretched position prevented the induction observed in soleus immobilized in a shortened position (i.e. mRNA expression of MAFbx and MuRF1 increased over 150% and 100%, respectively, in soleus immobilized in a shortened position when expressed relative to that of the soleus from the non‐immobilized hindlimb). These results suggest that fixed muscle length regulates atrogene expression whereby soleus immobilized in a stretched position prevents induction of atrogene expression observed in soleus immobilized in a shortened position.

## Discussion

The major finding of the present study is that fixed muscle length affects gene expression patterns in immobilized rat soleus. Though it has been shown that soleus immobilized in a stretched position does not exhibit disuse atrophy or attenutated rates of protein synthesis (Booth 1977; Goldspink 1977, 1978a; Loughna et al. 1986; Sasa et al. 2004), the present study investigated gene expression changes that might contribute to these observations. The results indicate that immobilization of soleus in a stretched as compared to a shortened position prevents induction of gene expression for the mTORC1 repressors, REDD1 and REDD2, which modulate mTORC1 signaling (Sofer et al. 2005; Miyazaki and Esser 2009). In addition, immobilization of soleus in a stretched as compared to a shortened position prevents induction of expression of atrogenes, which modulate rates of protein degradation (Bodine et al. 2001). Together, these findings help explain why soleus fixed in a stretched position does not exhibit disuse atrophy.

Recently, we identified an induction of REDD1 and REDD2 gene expression in soleus immobilized in a shortened position (Kelleher et al. 2013). This finding led to the development of a model in which immobilization of soleus in a shortened position induces REDD1 and REDD2, consequently leading to the attenuation of mTORC1 signaling and rates of protein synthesis, and to disuse atrophy. In the present study, we observed the same response in soleus immobilized in a shortened position, but a lack of REDD1 induction and suppression of REDD2 gene expression when soleus was immobilized in a stretched position. REDD1 and REDD2 are thought to repress mTORC1 signaling by acting through the TSC1·TSC2 complex (Inoki et al. 2003) to regulate Rheb (Ras‐homology enriched in brain) GTPase state. Rheb complexed with GTP is a direct activator of mTORC1 signaling, whereas Rheb complexed with GDP is not. Based on these results, we conclude that fixed muscle length modulates mTORC1 signaling at the level of REDD1 and REDD2 gene expression.

In accordance with other reports (Soares et al. 2007; Senf et al. 2008), the present study confirms that induction of atrogene expression is dependent on fixed muscle length. Senf and colleagues (Senf et al. 2008, 2010) observed induction of atrogene expression in rat soleus immobilized in a shortened position for 3 days. In contrast, Soares and colleagues (Soares et al. 2007) observed a suppression of atrogene expression in rat soleus immobilized for 1 and 2 days in a stretched position returning to non‐stretched expression levels after 4 days. We did not observe a suppression of atrogene expression after 3 days of immobilization of the soleus in a stretched position. Perhaps atrogene expression was suppressed at earlier time points and returned to nonimmobilized levels by the third day. Still, the results of the present study agree with both Senf et al. (2008) and Soares et al. (2007) whereby muscle immobilized in a stretched position prevented the induction of atrogene expression while muscle immobilized in a shorted position leads to the induction of atrogene expression.

Other studies have provided evidence to suggest that mTORC1 signaling is related to the extent to which a muscle is stretched. For example, acute passive stretch of skeletal muscle cells (Sasai et al. 2010) and tissue (Hornberger et al. 2004; Agata et al. 2009) enhances mTORC1 signaling. While some studies have shown an activation of Akt and signaling to the mTORC1 complex in response to stretch (Dogra et al. 2006; Agata et al. 2009; Sasai et al. 2010), Hornberger et al. (2004) suggested that acute passive stretch of skeletal muscle *ex vivo* stimulates mTORC1 signaling even in the absence of PI3K/Akt signaling. The latter suggestion would imply that muscle stretch stimulates mTORC1 signaling by an Akt‐independent mechanism (Hornberger et al. 2004). Gene expression is also influenced by skeletal muscle stretch whereby muscle immobilized in a stretched position exhibits repression of fast type and activation of slow myosin genes (Goldspink et al. 1992; Carson and Booth 1998a,b). Stretch and other mechanical stimuli can influence muscle gene expression through mechanosensory proteins in the muscle sarcomere (Linke and Kruger 2010). Proteins that bind the Z‐disk and M‐line relay mechanical strain information to cellular systems that control gene expression in the nucleus (Gautel 2011). For example, transcription factors and their interacting proteins bind to the muscle scaffolding protein, titin, and are released in response to increases in sarcomere length (Linke and Kruger 2010).

Evidence from human limb immobilization studies suggests that disuse atrophy manifests as a result of both attenuated rates of skeletal muscle protein synthesis in the basal, fasted condition, and the development of resistance to nutrient‐induced stimulation of skeletal muscle protein synthesis (a phenomenon termed “anabolic resistance”) (Glover et al. 2008; Phillips et al. 2009; Rennie 2009). According to protein balance calculations, approximately half of the deficit in muscle protein which causes disuse atrophy is attributed to anabolic resistance (Phillips et al. 2009). Recently, we reported that rat soleus immobilized in a shortened position exhibits resistance to leucine‐mediated stimulation of mTORC1 signaling (Kelleher et al. 2013). This resistance to stimulation of mTORC1 signaling by nutrients is likely to cause anabolic resistance to stimulation of protein synthesis and contribute to disuse atrophy. In the present study, all variables were measured only in a fasted state. Currently, the role of fixed muscle length in nutrient‐induced stimulation of mTORC1 signaling or rates of protein synthesis during hindlimb immobilization is unknown. Still, one might speculate that fixed muscle length modulates nutrient‐induced stimulation of protein synthesis. This speculation is based on the idea that if anabolic resistance contributes substantially to immobilization‐induced disuse atrophy, and no disuse atrophy is observed when muscle is immobilized in a stretched position, then muscle immobilized in a stretched position should be protected against the development of anabolic resistance.

## Conclusion

In conclusion, the results of the present study support the hypothesis that immobilization of soleus in a stretched position prevents changes associated with disuse atrophy such as induction of REDD1, REDD2, and atrogenes consequently leading to the attenuation of mTORC1 signaling and rates of protein synthesis as well as accelerated rates of protein degradation. Thus, fixed muscle length plays an important role in the regulation of atrophic gene expression.

## Acknowledgments

We thank Holly Lacko, Sharon Rannels, and Lauren Luongo for assistance in performing the studies.

## Conflict of Interest

The authors declare no conflicts of interest, financial or otherwise.
